# Large Conductance Ca^2+^-Activated K^+^ Channel (BK_Ca_) α-Subunit Splice Variants in Resistance Arteries from Rat Cerebral and Skeletal Muscle Vasculature

**DOI:** 10.1371/journal.pone.0098863

**Published:** 2014-06-12

**Authors:** Zahra Nourian, Min Li, M. Dennis Leo, Jonathan H. Jaggar, Andrew P. Braun, Michael A. Hill

**Affiliations:** 1 Dalton Cardiovascular Research Center, University of Missouri, Columbia, Missouri, United States of America; 2 Department of Medical Pharmacology and Physiology, University of Missouri, Columbia, Missouri, United States of America; 3 Department of Physiology, University of Tennessee Health Science Center, Memphis, Tennessee, United States of America; 4 Department of Physiology and Pharmacology, University of Calgary, Calgary, Alberta, Canada; The Chinese University of Hong Kong, Hong Kong

## Abstract

Previous studies report functional differences in large conductance Ca^2+^ activated-K^+^ channels (BK_Ca_) of smooth muscle cells (VSMC) from rat cerebral and cremaster muscle resistance arteries. The present studies aimed to determine if this complexity in BK_Ca_ activity may, in part, be due to splice variants in the pore-forming α-subunit. BK_Ca_ variants in the intracellular C terminus of the α-subunit, and their relative expression to total α-subunit, were examined by qPCR. Sequencing of RT-PCR products showed two α-subunit variants, ZERO and STREX, to be identical in cremaster and cerebral arteries. Levels of STREX mRNA expression were, however, significantly higher in cremaster VSMCs (28.9±4.2% of total α-BK_Ca_) compared with cerebral vessels (16.5±0.9%). Further, a low level of BK_Ca_ SS4 α-subunit variant was seen in cerebral arteries, while undetectable in cremaster arteries. Protein biotinylation assays, in expression systems and arterial preparations, were used to determine whether differences in splice variant mRNA expression affect surface membrane/cytosolic location of the channel. In AD-293 and CHO-K1 cells, rat STREX was more likely to be located at the plasma membrane compared to ZERO, although the great majority of channel protein was in the membrane in both cases. Co-expression of β1-BK_Ca_ subunit with STREX or ZERO did not influence the dominant membrane expression of α-BK_Ca_ subunits, whereas in the absence of α-BK_Ca_, a significant proportion of β1-subunit remained cytosolic. Biotinylation assays of cremaster and cerebral arteries showed that differences in STREX/ZERO expression do not alter membrane/cytosolic distribution of the channel under basal conditions. These data, however, revealed that the amount of α-BK_Ca_ in cerebral arteries is approximately 20X higher than in cremaster vessels. Thus, the data support the major functional differences in BK_Ca_ activity in cremaster, as compared to cerebral VSMCs, being related to total α-BK_Ca_ expression, regardless of differences in splice variant expression.

## Introduction

Potassium channels play an important role in the regulation of VSMC membrane potential and contractile activity. In particular, large conductance Ca^2+^-activated, K^+^ channels (BK_Ca_) are activated in response to membrane depolarization and increases in intracellular Ca^2+^ to affect membrane hyperpolarization [1], [2]. While BK_Ca_ channels are widely expressed in both electrically excitable and non-excitable cells [3], [4], they are relatively abundant in smooth muscle and play a key role in the regulation of vascular tone [5], [6]. Structurally, the functional BK_Ca_ channel exists as a tetramer of α-subunits forming the ion channel pore together with tissue specific auxiliary β-subunits (β1–β4) which are typically present in a 1∶1 stoichiometry [7]. The BK_Ca_ α-subunit consists of seven transmembrane spanning domains (S0–S6) including the extracellular N-terminus, P-loop between S5 and S6 domains, and a large intracellular C terminus containing a number of regulatory sites including the regulators of conductance for K^+^ (RCK 1 and 2) and 2–3 Ca^2+^ binding sites.

**Figure 1:**
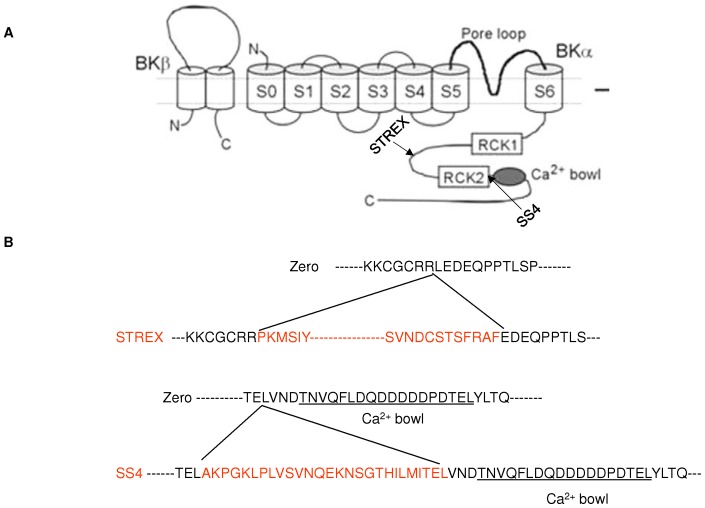
Location and amino acid sequences of ZERO and STREX splice variants of BK_Ca_ α-subunit. **S**chematic diagram illustrating (A) sites of STREX and SS4 splicing variants of α-BK_Ca_ and (B) the amino acid sequences of the splicing inserts (Adapted from reference [5]).

The BK_Ca_ α-subunit is encoded by a single gene (KCNMA1) containing 27 distinct exons, in contrast to each β-subunit, which is encoded by four distinct exons [8]. BK_Ca_ channels appear to achieve part of their functional diversity through alternative pre-mRNA splicing of the KCNMA1 gene [9], [10]. Up to ten alternative splicing sites have been identified for the vertebrate BK_Ca_ α-subunit [11]. Most variation occurs in the intracellular C-terminal part in the linker region between domains RCK1 and RCK2 and upstream of the “calcium bowl” [12]. Alternative splicing can modify the functional properties of BK_Ca_ channels, including Ca^2+^ and voltage sensitivity, cell surface expression, and regulation by diverse intracellular signaling pathways. One of the most thoroughly studied α-BK_Ca_ splice variants is the STREX exon (STRess axis regulated EXon), which derives its name from its splicing regulation by stress-axis hormones [13]. It has been shown that the STREX exon (an insertion of 58 amino acids in the C-terminal splice site 2 of the α-subunit protein) confers distinct functional phenotypes onto BK_Ca_ channels, such as altered Ca^2+^ sensitivity and changing responsiveness of channels to cAMP signaling from stimulatory to inhibitory, compared with the ZERO variant, lacking this insert [9], [14]–[17]. It has also been shown that BK_Ca_ channels containing the SS4 splice variant (an insertion of a 27 amino acid segment upstream to the C-terminal Ca^2+^-bowl in splice site 4 of the α-subunit) were activated more rapidly than the ZERO variant in the presence of the same voltage stimulus, and the difference in these activation kinetics was dependent on the concentration of intracellular Ca^2+^ [18], (also see Figure 1).

Alternatively, it has been reported that intracellular trafficking of α-BK_Ca_ may be one of the main post-translational modifications that can regulate the number of ion channels at the cell surface [19]. This mode of regulation can also be modulated by accessory β-subunits. While limited studies have addressed the effects of α-BK_Ca_ splice variants on channel trafficking to plasma membrane [20]–[24], there is some discrepancy in the reported findings. For example, Kim et al (2007) reported the expression of two α-BK_Ca_ splice variants, termed VEDEC and QEDRL, in chick ciliary ganglion neurons that differ at the extreme C-terminus. Using HEK293T and NG108-15 cells and a cell surface biotinylation assay, QEERL channels showed markedly higher levels of constitutive expression of α-BK_Ca_ at the plasma membrane compared with VEDEC channels, which tend to remain in the cytosol [21]. The same group further showed that co-expression of avian β1-subunits with the VEDEC isoform α-BK_Ca_, prevented the inhibitory effect of the VEDEC sequence on cell surface expression [20]. In contrast, studies from Toro and colleagues (2006) showed that co-expression of human β1-subunit with a human pore-forming α-subunit enhanced internalization of the α-subunit [25].

Previous electrophysiological studies from our laboratory have demonstrated that BK_Ca_ channel activity differs significantly in VSMCs from cremaster muscle arteries compared with cerebral arteries. In particular, our functional data have revealed a decreased Ca^2+^ sensitivity of cremaster BK_Ca_ channels, resulting in more positive levels of Em being required in cremaster VSM cells to generate similar levels of outward K^+^ conductance [26]. Similarly, Jackson and Blair (1998) described cremaster muscle BK_Ca_ channels as being normally ‘silent’, but suggested that their activity could be ‘recruited’ during vasoconstriction [27]. Therefore in this study, we first hypothesized that the functional differences between these two resistance vasculatures may be due, in part, to the expression of splice variants of the BK_Ca_ α-subunit. We chose to focus on STREX and SS4 splice variants, as splice sites where they are located (i.e. 2 and 4) contain regulatory phosphorylation, palmitoylation and Ca^2+^ interaction sites that could functionally impact channel activity. We further hypothesized that an additional influence of α-subunit splice variation may be on the surface membrane location of the channel and whether this could be affected by the accessory β1-subunit.

## Material and Methods

### Tissue isolation and vessel RNA purification

All experiments and protocols were approved by the Animal Care and Use Committee, University of Missouri, USA. Our studies used male Sprague-Dawley rats weighing between 180–280 g. Rats were anaesthetized with sodium pentobarbital (Nembutal, 100 mg/kg body weight) given by an intraperitoneal injection. Cremaster muscles were surgically removed, as previously described [28], and placed in a cooled (4°C) dissection chamber. Following sacrifice by anesthetic overdose, a craniotomy was performed and the brain was removed intact and similarly placed in a cooled dissection chamber.

First- and second-order arterioles (1A/2A) from cremaster muscle and mid-cerebral arteries were isolated and rapidly subjected to total RNA purification using a Melt Total Nucleic Acid isolation system kit (Life Technologies, Carlsbad, CA, USA) following the manufacturer's instructions. All samples were treated by TURBO DNase digestion (Life Technologies) to minimize contamination with genomic DNA. The concentration and purity of RNA for each sample was determined by UV absorbance using a Nanodrop ND-1000 spectrophotometer (Thermo Scientific, Rockford, IL, USA) and samples were stored at −80°C until conversion to cDNA. Equal amounts of total vessel RNA extract were then reverse-transcribed into a single strand cDNA using a Superscript III First-Strand synthesis system (Life Technologies) according to the manufacturer's instructions.

### Real-time quantitative PCR

Real-time PCR was performed in triplicate, in 96-well plates, on cDNAs prepared from each sample (n = 4–5) using KAPA SYBER FAST qPCR Kit Master mix (KAPA Biosystems, Woburn, MA, USA). PCR was performed using a Mastercycler EP Realplex^2^ (Eppendorf-North America, Westbury, NY, USA). Reaction volume/well contained 20 µl∶10 µl of master mix, 1 µl of each sense and antisense primers (5 µM), 1 µl of cDNA template and the remainder DNase-free water. Primers used in this study were based on previously published papers: ZERO [26] STREX [9], and β-actin [29]. Primers for SS4 variant were designed using Real-Time PCR primer software from Integrated DNA Technologies (IDT). Details of oligo-DNA primers used to amplify BK_Ca_ α-subunits (STREX, SS4) and ZERO variants, accession numbers for the template sequences and the expected product sizes are shown in Table 1. ZERO variant primers were designed in regions of transmembrane domains in which no splice variant existed, and its expression was utilized as an indication of the total expressed α-subunit mRNA [9], [13].

**Table 1:** Sequence of primers used for end-point and real-time PCR.

	Accession number	Primer sequence	Amplicon length	Amplification efficiency
End-point PCR				
α-BK_Ca_	NM_031828	F: TACTGCAAGGCCTGTCATGATG	342	
		R: TCATCAGCTTCGGGGATGTGTT		
Real-time PCR				
STREX	NM_031828	F: TTTGATTGCGGACGTTCTGA	77	2.063
		R: TCTCTCAAGGGTGTCCACGTTAC		
SS4	AF_135265	F: CAAGTTGCCTTTGGTATCAGTC	131	2.013
		R: GCTCTGTGTCAGGGTCATC		
ZERO	NM_031828	F:AAACAAGTAATTCCATCAAGCTGGTG	137	2.006
		R: CGTAAGTGCCTGGTTGTTTTGG		
β-actin	NM_031144	F: CCTCTATGCCAACACAGTGCTGTCT	128	1.993
		R: GCTCAGGAGGAGCAATGATCTTGA		

(F: forward primer, R: reverse primer).

Real-time PCR protocols were performed as follows: pre-heating at 95°C for 2 min, 40 cycles of two-step cycling of denaturation at 95°C for 3 sec and annealing/extension steps of 25 sec at 58°C. For each qPCR determination, no enzyme and no template conditions were included to test for contamination of assay reagents. An arbitrary rat mid-cerebral artery cDNA sample was included in each plate to provide a constant calibrator point for all samples. After the final PCR cycle, a melting curve analysis was routinely performed to identify the presence of primer-dimers and to analyze the specificity of the reaction. Data were collected and analyzed using Realplex software (Eppendorf-North America). The amplification efficiencies between targets and housekeeping genes (i.e. β-actin) were initially verified to be approximately equal (Table 1), allowing the comparative threshold (Ct) method for quantification to be used [30]. The relative expression level (R) was calculated with equations as follows: R = 2^−ΔΔCt^ = 2^−(ΔCt sample − ΔCt calibrator)^ for the target genes in each sample set according to the published 2^−ΔΔCt^ method [30]. Changes in mRNA expression levels were calculated from an average of triplicate measurements and are reported as fold changes relative to the ZERO variant, after normalization to β-actin. Data were analyzed using an unpaired student *t*-test: a statistically significant difference was assumed at P≤0.05.

### Cell surface Biotinylation assay on cultured cells

Plasmid constructs containing cDNA for full-length rat BK_Ca_ ZERO variant or BK_Ca_ STREX variant (gifts from Dr. Michael J. Shipston) were transiently transfected into AD-293 cells (240085, Agilent Technology, Santa Clara, CA, USA) or CHO-K1 cells (CCL-61, ATCC, Manassas, VA, USA) with FuGENE 6 Transfection Reagent (Roche Diagnostics, Indianapolis, IN, USA). A bovine β1-BK_Ca_ plasmid DNA was also used in some experiments as its sequence shares high homology (>95%) with rat β1-BK_Ca_ channel protein. Cell surface biotinylation assays were performed 24–48 hours post-transfection. In brief, live transfected cells were washed three times with Hanks' buffered salt solution (HBSS) and then incubated on ice for 2 hours in the presence of a freshly prepared 0.5 mg/ml mixture of biotinylation reagents, EZ Link Sulfo-NHS-Lc-Lc-Biotin (21338, Thermo Scientific) and EZ Link Maleimide-PEG-Biotin (21901, Thermo Scientific). Total protein was determined to allow normalization for Avidin pull-down of biotinylated proteins after quenching of biotinylation process by ice-cold 100 mM glycine in HBSS (3x in 1 min interval incubation). Biotinylated cells were homogenized in RIPA buffer plus 1% protease inhibitor cocktail (Sigma-Aldrich, St. Louis, MO, USA), incubated on ice (30 min) and sonicated for 45 sec. Cellular debris was removed by centrifugation at 6,000 g for 10 min at 4°C. Total protein concentration was determined using the BCA protein assay kit (Thermo Scientific). Equal amounts of total biotinylated cell lysates were subsequently incubated with Monomeric Avidin Agarose (20228, Thermo Scientific) overnight at 4°C, followed by washing with cold HBSS (3x) and one time with water. Finally, the cytosolic fractions of cells transfected with either α-BK_Ca_ splice variants or β1-BK_Ca_ subunit were separated from biotinylated cell surface proteins by centrifugation (11,000 g/2 min/4°C). The biotinylated membrane proteins were then eluted from the beads by heating at 45°C/15 min in 2× Laemmli protein sample buffer [31]. Isolated cell surface and cytosolic proteins were separated by SDS-PAGE on 4–20% TGX Precast Gels (Bio-Rad, Hercules, CA, USA), transferred onto polyvinylidene difluoride membranes and probed with a mouse monoclonal anti-BK_Ca_ channel (clone L6/60, 1∶500, NeuroMab, Davis, CA, USA) or an anti-BK_Ca_ β subunit antibody (ab3587, 1∶500, Abcam, Cambridge, MA, USA). Bound antibody was detected using SuperSignal West Dura ECL Chemiluminescent Substrate (34075, Thermo Scientific). Images were collected using a ChemiDoc XRS+ System (Bio-Rad) and analyzed by Image Lab software. Parallel control biotinylation assays were conducted with mock transfected cells and cells with streptavidin beads in the absence of biotin incubation. In mock transfected cells, no bands were detected related to the α-BK_Ca_ splice variants (ZERO, STREX) or the β1 subunit (data not shown). These control studies confirmed the absence of endogenous BK_Ca_ channels in CHO-K1 cells and the specificity of the antibodies used in this study.

### Cell Surface Biotinylation assay on vessels

Biotinylation of surface proteins in intact cerebral and cremaster arteries was performed to detect the cell surface membrane expression of native α-BK_Ca_ channels in these vessel types. To have adequate amounts of total protein, first- and second-order cremaster arterioles from four male Sprague-Dawley rats (180–280 g) were pooled together for each separate experiment. In parallel, the whole Circle of Willis vasculature was isolated from two animals, cleaned of connective tissue and pooled to provide a cerebral artery sample. Arteries were incubated in a freshly prepared 1 mg/ml mixture of Biotin reagents, as above, in whole cell buffer solution (in mM 10 HEPES, 9 Glucose, 6 KCl, 134 NaCl, 2 CaCl_2_.2H2O, and 1 MgCl_2_.6H2O) for one hour at room temperature while undergoing constant horizontal shaking. The arteries were then incubated at room temperature with quenching solution of 100 mM glycine in PBS for 15 min to remove any unbound biotin. The biotinylated vessels were homogenized to prepare total protein as previously described [32]. Equal amounts of total protein (∼50–60 µg) were incubated with Monomeric Avidin Agarose. After one hour of avidin incubation at room temperature [33], [34], the non-biotinylated (cytosolic) protein fraction was separated from the biotinylated (cell membrane) protein fraction by centrifugation at 11,000 g/2 min/4°C. Biotinylated surface proteins were eluted from the avidin beads by boiling for 3 min in 2× Laemmli buffer containing β-mercaptoethanol (5% v/v). Western blot analysis of surface and cytosolic proteins was performed using mouse anti α-BK_Ca_ channel (1∶500, NeuroMab) or anti-BK_Ca_ β subunit (1∶500) primary antibodies. Quantification of cell surface and cytosolic protein bands was analyzed using Image Lab software (Bio-Rad) and are expressed as percentage of total protein.

## Results

### Identification of α-BK_Ca_ splice variants by end-point PCR

**Figure 2:**
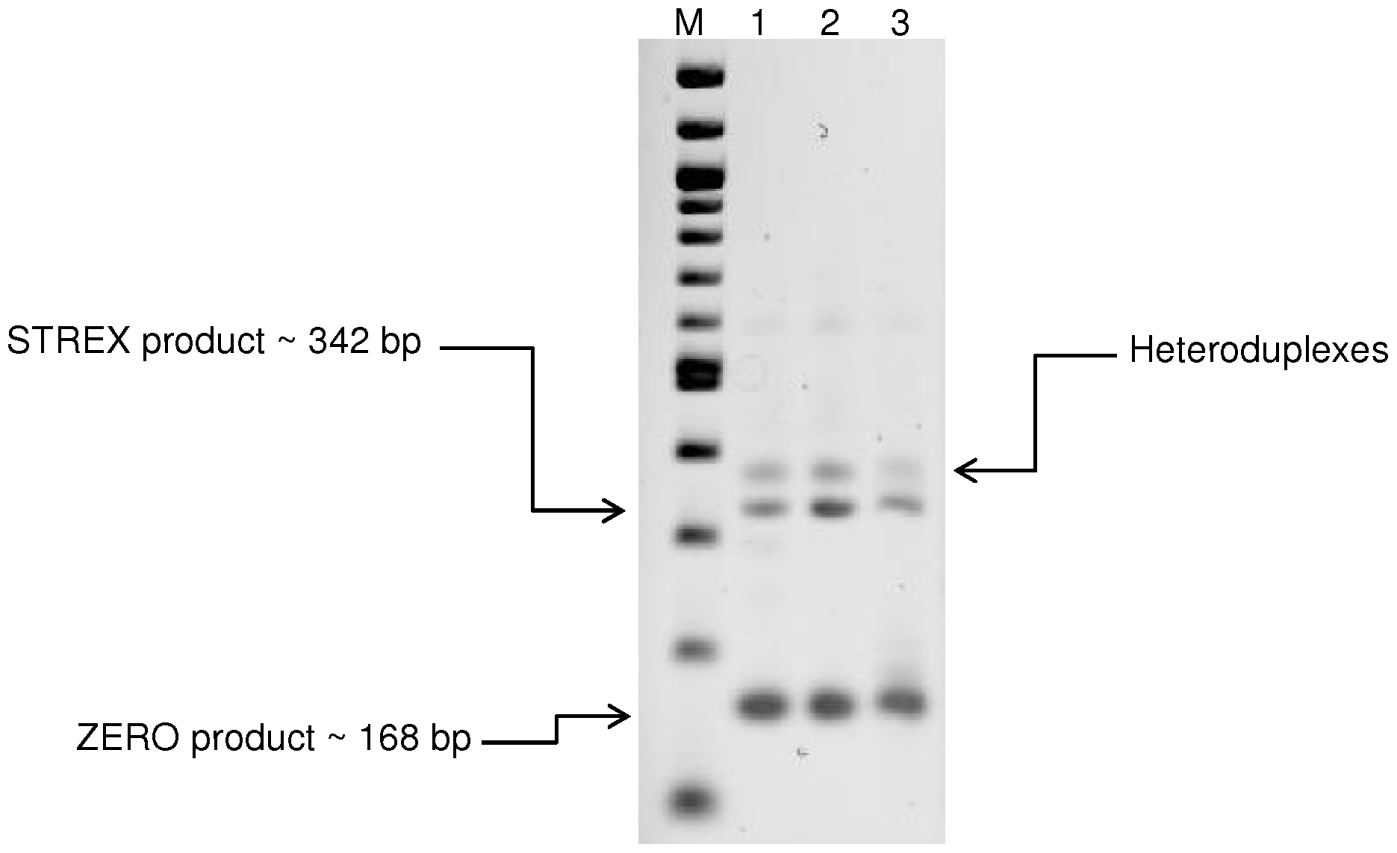
Detection of ZERO and STREX variants in rat cerebral and cremaster arteries. End-point PCR products generated from cDNA derived from rat total RNA transcripts, with and without the STREX exon. Size markers are shown in the lane marked as M. Additional lanes display PCR products detected in testis (lane 1, included as a positive control), mid-cerebral arteries (lane 2) and cremaster arterioles (lane 3). Results are representative of n = 3 separate experiments.

For initial identification of STREX and ZERO variants, end-point PCR was performed using primers designed to amplify the alternative splice site 2 (See Table 1 for details). Testis cDNA was used as a positive control [11]. PCR products of three separate experiments from different experimental animals were analyzed by electrophoresis and subsequently verified by sequencing. As shown in Figure 2, two dominant bands were detected in both vasculatures. The lower band with predicted size of 168 bp was determined to be ZERO variant (α-subunit without splice insert) by direct product sequencing and the upper band (∼342 bp) was confirmed to be STREX variant (α-subunit with the insertion of 174 bp at splice site 2). The third visible band likely constitutes heteroduplexes between sense and antisense strands of STREX and ZERO products consistent with earlier reports [35].

### Quantification of α-BK_Ca_ splice variants by qPCR

**Figure 3:**
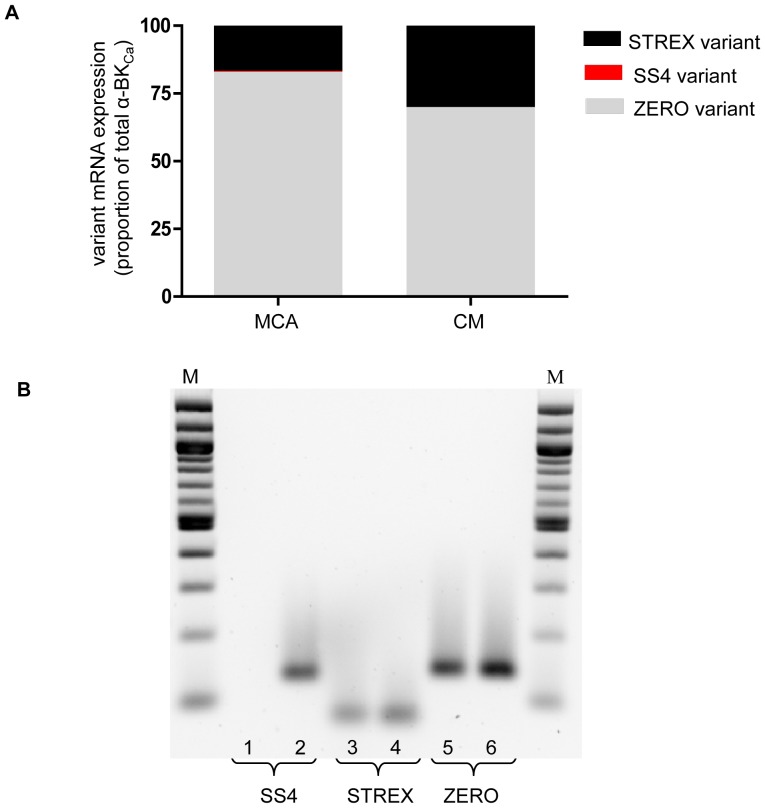
QPCR of BK_Ca_ α-subunit splice variants in rat cerebral and cremaster arteries. (A) Relative mRNA expression levels for STREX, SS4 and ZERO variants calculated as a percentage of total α-BK_Ca_ mRNA detected in mid-cerebral arteries and cremaster arterioles. (B) QPCR products of SS4, STREX and ZERO variants of α-BK_Ca_ subunit as separated on a 2% agarose gel. Size markers shown in the lane denoted M. QPCR products generated from cremaster arterioles are depicted in lanes 1, 3, 5 and products from mid-cerebral artery are shown in lanes 2, 4, and 6. Real-time PCR results are shown for n = 4–5 samples of each vascular bed performed in triplicate.

Identification of the SS4 variant was performed by qPCR together with subsequent quantification of expression levels of STREX variant relative to ZERO using a further set of primers (Table 1). As shown in Figure 3A and B, while a very low level of SS4 was detected in mid-cerebral arteries (0.42±0.1% of total α-BK_Ca_), the variant was undetectable in cremaster vessels. A higher level of expression of the STREX variant was detected in cremaster arteries (28.9±4.2% of total α-BK_Ca_) compared to mid-cerebral (16.5±0.9% of total α-BK_Ca_) arteries (P<0.05). Thus, ZERO variant was calculated to be significantly (P<0.05) greater in mid-cerebral (83.1±0.9% of total α-BK_Ca_) compared to cremaster (71.1±4.2%) arteries (Figure 3A).

### Cell Surface expression of BK_Ca_ ZERO or STREX variants in expression systems

**Figure 4:**
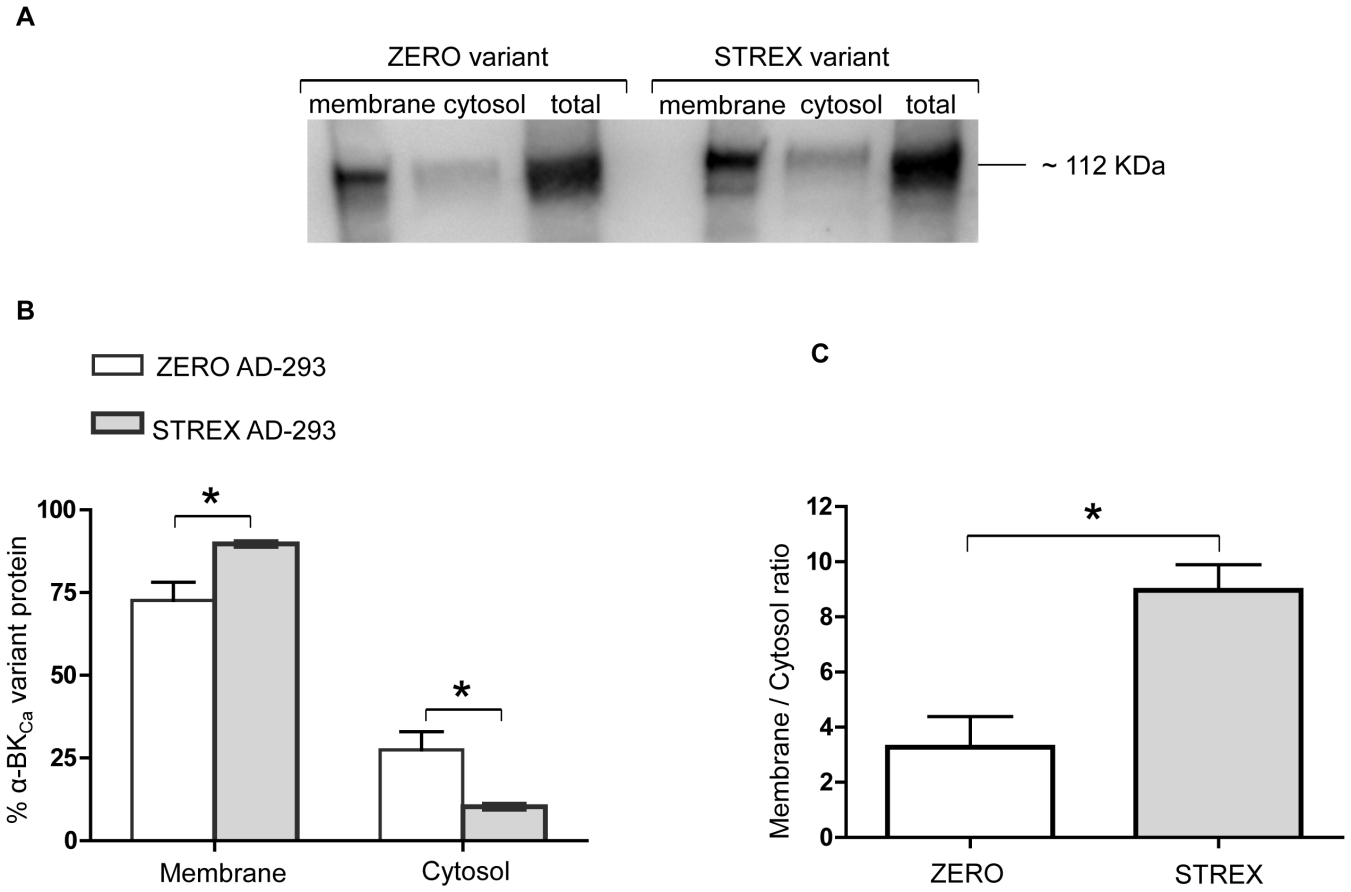
The cell surface and cytosolic expression of BK_Ca_ α-subunit splice variants in cultured cells. Using an AD-293 cell culture system, α-BK_Ca_ STREX variant shows a significantly higher cell surface expression compared with the ZERO variant. Corresponding changes in the cytosolic fraction are observed. (A) Representative Western blot showing membrane, cytosolic, and total levels of α-BK_Ca_ protein in AD-293 cultured cells, transfected with either α-BK_Ca_ splice variants ZERO or STREX. (B) Summary of membrane vs cytosolic distribution of transfected ZERO or STREX variants of α-BK_Ca_ channel protein (expressed as % of total α-BK_Ca_ protein). (C) Membrane to cytosolic expression ratio of ZERO and STREX variants in transfected cultured AD-293 cells. Results are shown for n = 4 separate experiments and are presented as mean ± SEM (*P<0.05, unpaired t-test).

To investigate the cell surface location of α-BK_Ca_ splice variants in a cell culture system, equal amounts of full-length cDNAs of rat ZERO or STREX variants were initially transfected into AD-293 cells (Figure 4A). As shown in Figure 4B, although both variants were predominantly targeted to the cell surface, the STREX variant of α-BK_Ca_ shows a significantly higher level of cell surface expression (P = 0.02, unpaired t-test) than the ZERO variant. Conversely at the cytosolic level, the ZERO variant shows a significantly higher level of expression as compared with STREX (P = 0.02, unpaired t-test). The ratio of membrane to cytosol expression was also significantly higher for the STREX variant than ZERO (Figure 4C).

**Figure 5:**
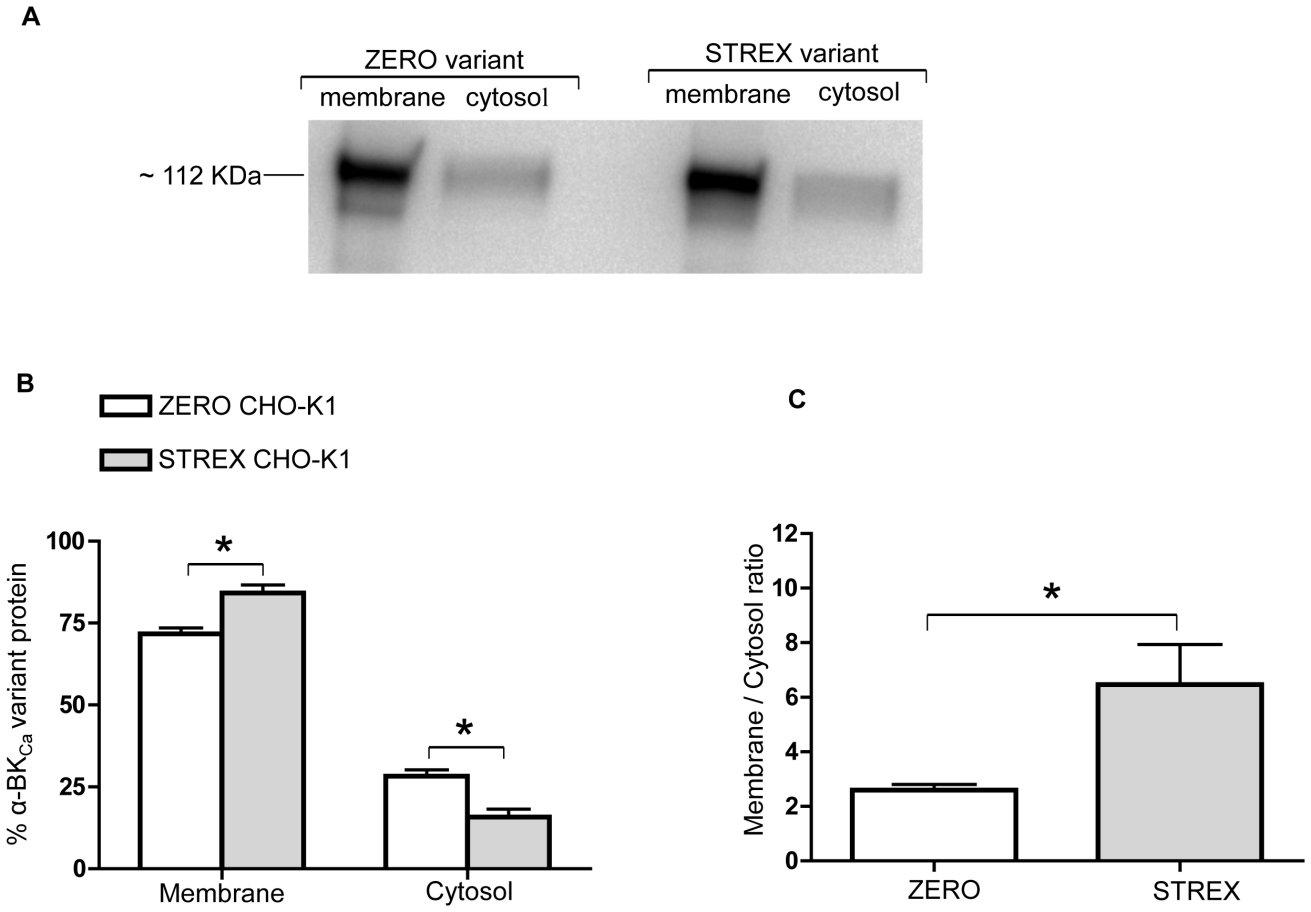
Membrane and cytosolic expression of ZERO and STREX variants in CHO-K1 cells. In a CHO-K1 cell culture system, the α-BK_Ca_ ZERO variant shows significantly lower cell surface expression than the STREX variant. Corresponding changes in the cytosolic fraction are observed. (A) Representative Western blot showing surface and cytosolic levels of α-BK_Ca_ protein in CHO-K1 cell culture system, transfected with either α-BK_Ca_ splice variants ZERO or STREX. (B) Summary of membrane vs cytosolic expression of transfected ZERO or STREX variants of α-BK_Ca_ channel protein (expressed as % of total α-BK_Ca_ protein). (C) Membrane to cytosolic expression ratio of ZERO and STREX α-BK_Ca_ proteins in transfected cultured CHO-K1 cells. Results are shown for n = 6 separate experiments and are presented as mean ± SEM (*P<0.05, unpaired t-test).

To confirm that this trafficking pattern was not cell dependent, similar experiments were conducted using CHO-K1 cells (Figure 5A). As illustrated in Figure 5B, the STREX variant again shows increased distribution at the cell membrane compared with ZERO (P = 0.002, unpaired t-test). The cytosolic expression of STREX in CHO-K1 cells shows significantly lower expression than the ZERO variant, similar to that observed in the AD-293 cell culture system. As shown in Figure 5C, the ZERO variant shows a significantly lower ratio of membrane to cytosolic expression in CHO-K1 cells. Since these two α-BK_Ca_ subunit splice variant exhibit the same expression pattern in AD-293 and CHO-K1 cells, we used CHO-K1 cells as our cell system model for future experiments.

### Cell Surface location of co-transfected BK_Ca_ ZERO or STREX variants with β1-subunit in CHO-K1 cells

**Figure 6:**
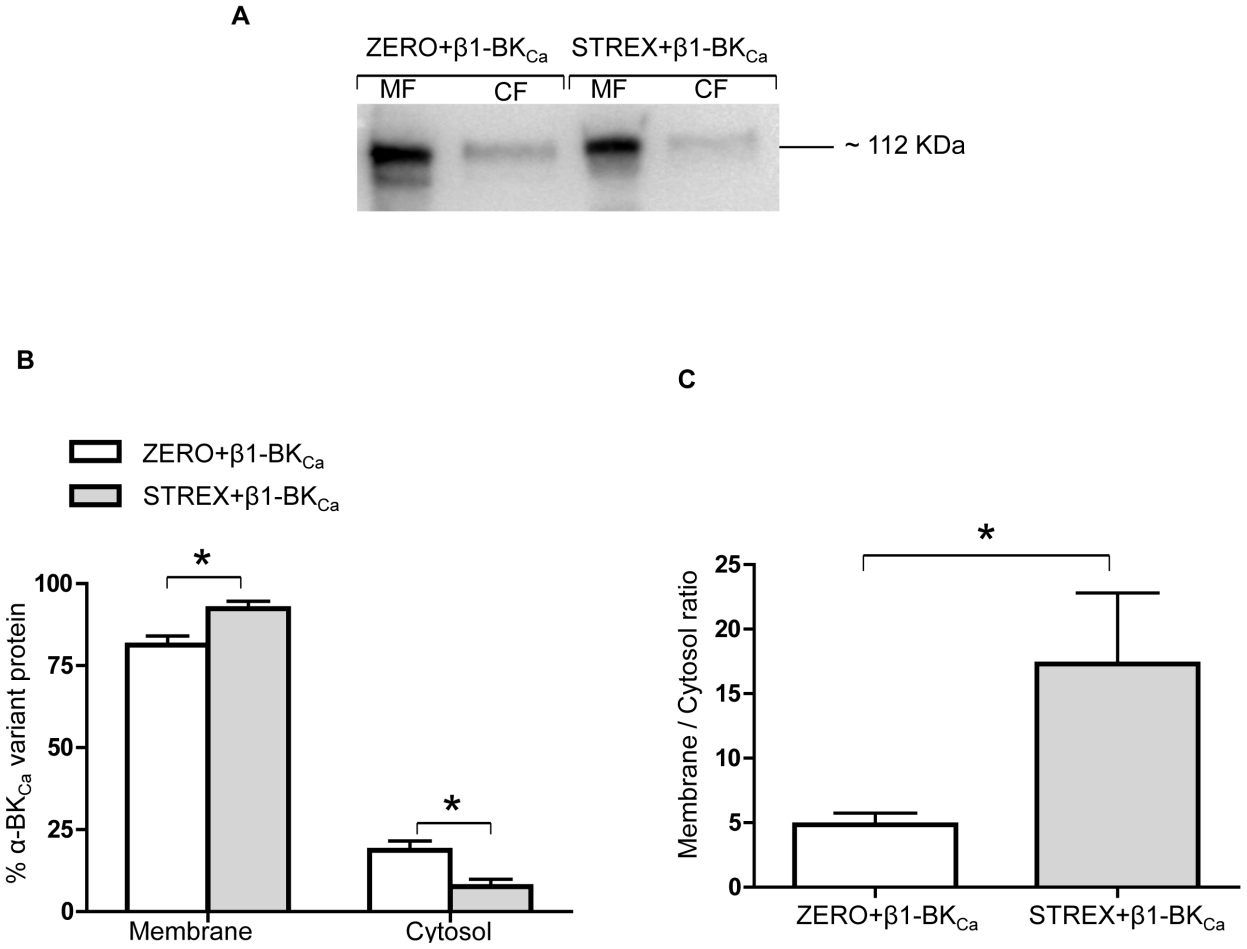
The effect of β1-BK_Ca_ subunit on cell surface expression of α-BK_Ca_ splice variants. Co-transfection of β1-BK_Ca_ subunit has little apparent impact on cell surface expression of α-BK_Ca_ ZERO or STREX variants. (A) Representative Western blot showing levels of membrane (MF), and cytosolic (CF) fractions of α-BK_Ca_ protein in CHO-K1 cultured cells, transfected with either α-BK_Ca_ splice variants ZERO or STREX. (B) In the CHO-K1 cell culture system, α-BK_Ca_ STREX variant shows dominant cell surface expression when co-transfected with β1-BK_Ca_. Results are shown for n = 5 separate experiments and are presented as mean ± SEM (*P<0.05, unpaired t-test). (C) Membrane to cytosolic ratio for the expression of ZERO or STREX proteins when co-transfected with β1-BK_Ca_ in cultured CHO-K1 cells. Results are shown for n = 5 separate experiments and are presented as mean ± SEM (* P = 0.05, unpaired t-test).

As holo-BK_Ca_ channels in resistance arteries contain both α- and β1-BK_Ca_ subunits, we hypothesized that co-expression of β1-subunit in CHO-K1 cells may equalize the cell surface levels of the two variants. To investigate the impact of β1-BK_Ca_ subunit on cell surface expression of α-BK_Ca_ splice variants ZERO and STREX, β1-BK_Ca_ subunit was co-transfected with either the ZERO or STREX variant (Figure 6A). In the presence of β1-BK_Ca_ subunit, the α-subunit variants continued to be predominantly located in the plasma membrane with the STREX variant present at higher levels than ZERO (Figure 6B). This resulted in the same relative expression pattern (Figure 6C) as seen when the α-BK_Ca_ splice variants were expressed without the β1-BK_Ca_ subunit (Figure 5C). These data could indicate a dominant cell surface expression pattern of α-BK_Ca_ splice variants of ZERO and STREX in cell culture systems.

**Figure 7:**
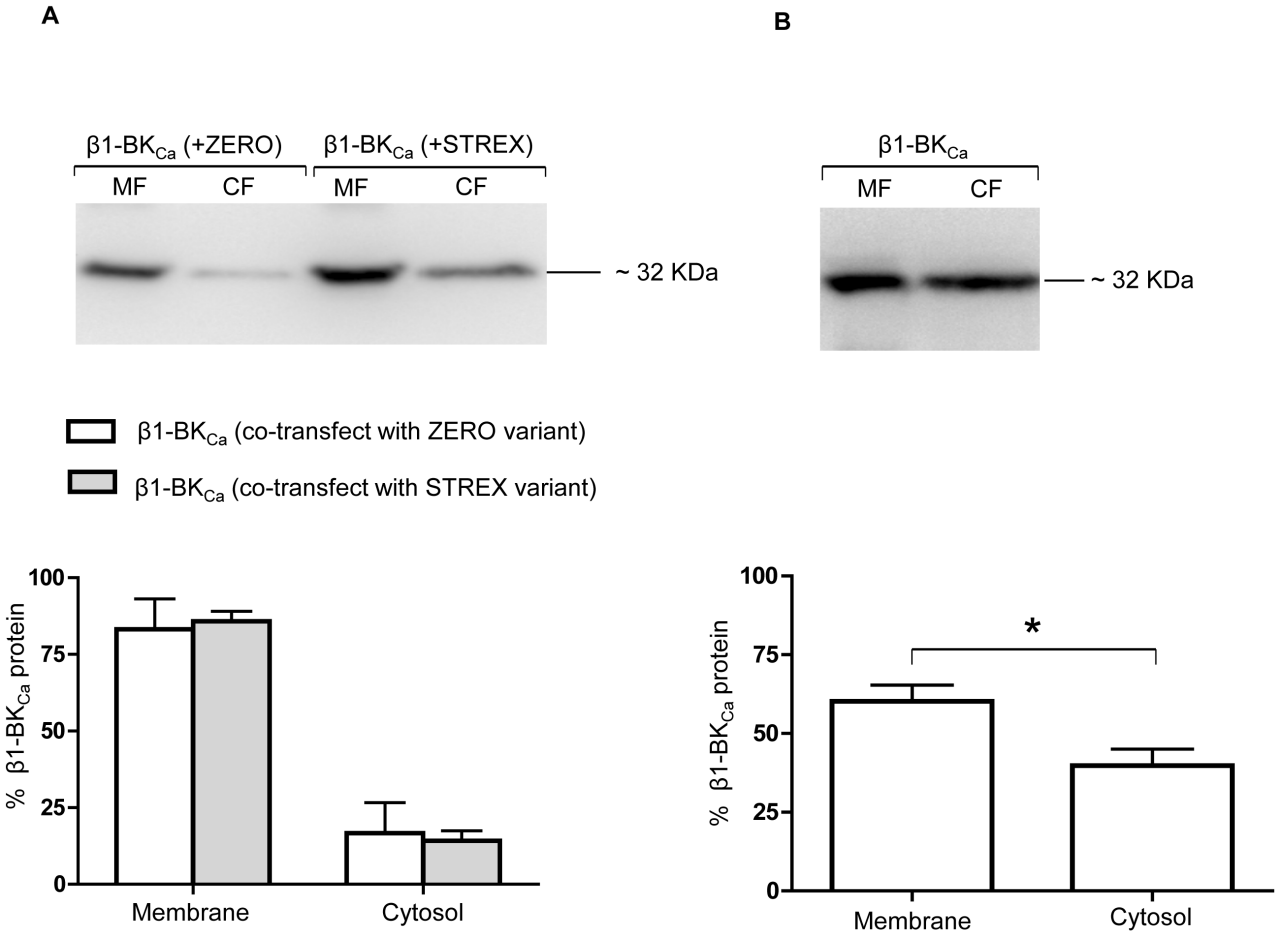
Cell surface expression of β1-BK_Ca_ subunit in the presence and absence of α-BK_Ca_ splice variants. Co-transfection of ZERO or STREX variants with β1-BK_Ca_ subunit enhances cell surface trafficking of β1-BK_Ca_ subunit. The top image in panel (A) represents a Western blot for membrane (MF) and cytosolic (CF) fractions of β1-BK_Ca_ protein in CHO-K1 cultured cells, co-transfected with either α-BK_Ca_ splice variants ZERO or STREX. The bottom Figure in panel (A) shows the membrane vs cytosolic expression of transfected β1-BK_Ca_ channel protein when co-transfected with either α-BK_Ca_ splice variants of ZERO or STREX. Results are shown for n = 5 separate experiments. The top image in panel (B) shows a representative Western blot of membrane (MF) and cytosolic (CF) fractions of β1-BK_Ca_ protein in CHO-K1 cultured cells, in the absence of either α-BK_Ca_ splice variants ZERO or STREX. The bottom Figure in panel (B) shows membrane vs cytosolic expression of β1-BK_Ca_ subunit in cultured CHO-K1 cells in the absence of the α-BK_Ca_ splice variants ZERO or STREX. Results are shown for n = 6 separate experiments and are presented as mean ± SEM (*P<0.05, unpaired t-test).

To determine the effect of ZERO or STREX variants on the cellular distribution of β1-BK_Ca_ subunits, biotinylated β1-BK_Ca_ subunit proteins were also probed in cells co-transfected with β1-BK_Ca_ and either the ZERO or STREX variant. As shown in Figure 7A, a high level of cell surface expression of β1-BK_Ca_ subunit (84.7±4.4%) was observed when co-transfected with either the ZERO or STREX variant. To assess whether over-expression of β1-BK_Ca_ subunit, alone, is sufficient to stimulate its surface trafficking, CHO-K1 cells were transfected with only β1-BK_Ca_ cDNA and β1-BK_Ca_ subunit surface location assessed by the cell-surface biotinylation assay. Under these conditions, as shown in Figure 7B, the cell surface labeling of β1-BK_Ca_ subunit showed a significant decrease (P = 0.01) to approximately 60.2±5.2%, which was accompanied by a significant increase in cytosolic levels of β1-BK_Ca_ subunit from approximately 15.3±4.5% to 39.8±5.2%. These data appear to indicate a stimulatory effect of α-BK_Ca_ splice variants on the surface trafficking of the regulatory subunit β1-BK_Ca_, while there was no apparent impact on the surface membrane location of either ZERO or STREX by β1-BK_Ca_ subunit.

### Cell surface expression of total α-BK_Ca_ protein in cerebral vs cremaster arteries

**Figure 8:**
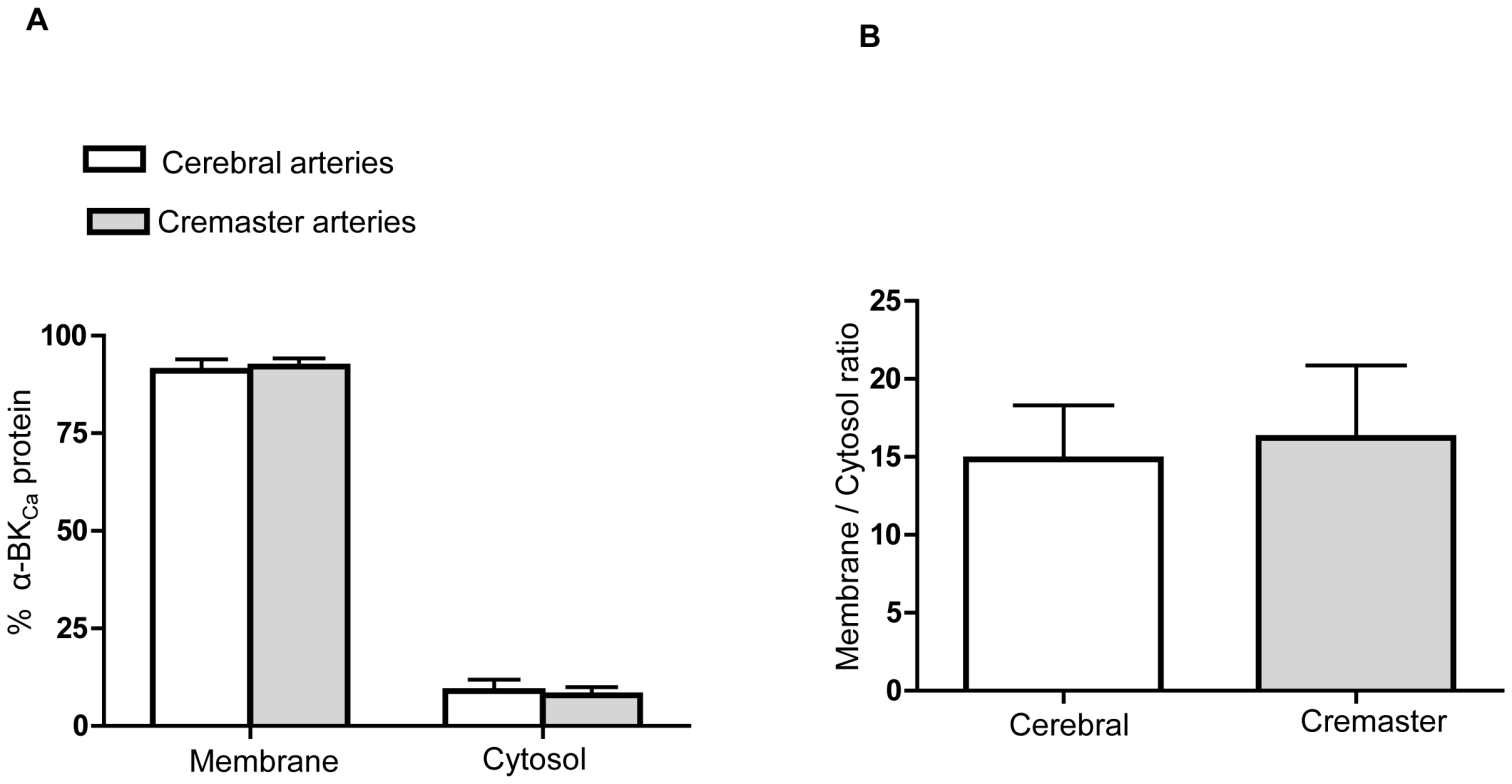
Membrane and cytosolic expression of α-BK_Ca_ subunits in cerebral vs creamster arteries. α-BK_Ca_ channels are predominantly located at the cell surface of VSMCs in both rat cerebral and cremaster arteries under basal conditions. (A) Cell surface α-BK_Ca_ proteins were determined using the biotinylation assay from whole cerebral (Circle of Willis pooled from 2 animals in each experiment) and cremaster arteries (first- and second-order cremaster arterioles pooled from 4 animals in each experiment). Results are shown for n = 6 separate experiments for cerebral and n = 5 for cremaster arteries. Results are shown as mean ± SEM. (B) Membrane to cytosolic ratio of α-BK_Ca_ channels in cerebral vs cremaster arteries (the corresponding Western blot showing cell surface and cytosolic levels of α-BK_Ca_ protein is shown in Figure 9A).

**Figure 9:**
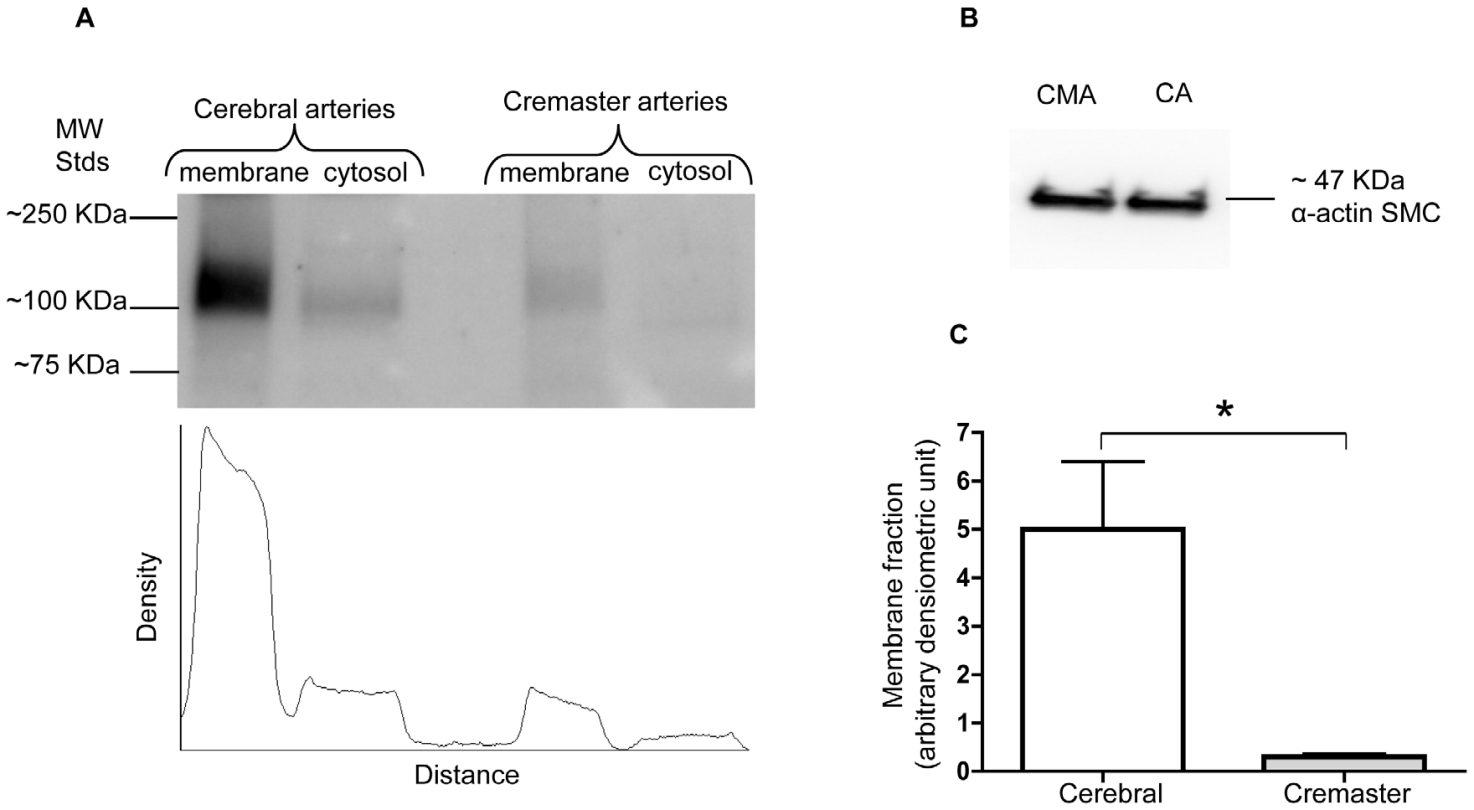
Quantification of cell surface expressed α-BK_Ca_ channels in cerebral vs cremaster arteries. Total amount of membrane α-BK_Ca_ channel in cerebral arteries is approximately 20 times higher than that in cremaster arterioles. The Western blot in panel (A) shows a representative experiment depicting the distribution of α-BK_Ca_ protein in membrane and cytosolic fractions prepared from cerebral arteries and cremaster arterioles. The scan beneath the blot quantifies the intensity of the α-BK_Ca_ protein immunoreactive band in each sample, as detected by densitometry. The Western blot in panel (B) shows a loading control in which 2 µg of total protein for both cerebral arteries (CA) and cremaster arterioles (CMA) was probed with α-actin SMC antibody. Panel (C) shows group data for n = 5 experiments. Results are shown as mean ± SEM (*P<0.05, unpaired t-test).

Previous electrophysiological studies from our laboratory have demonstrated that BK_Ca_ channel activity differs significantly in VSMCs from cremaster muscle arteries compared with cerebral arteries [26]. Our functional data show a decreased Ca^2+^ sensitivity of cremaster VSMC BK_Ca_ channels compared with those of cerebral arteries, resulting in more positive levels of Em being required for cremaster VSMCs cells to generate similar levels of outward K^+^ current. As a result, we hypothesized that these functional differences in channel activity could arise from differences in the molecular configuration of the channel in the two VSMC types affecting channel properties such as cell surface trafficking of α-BK_Ca_ protein. Although the lack of specific available antibodies to distinguish ZERO from STREX variants is a technical limitation to detect α-BK_Ca_ splice variants at the protein level, the biotinylation assay was used to determine the extent of α-BK_Ca_ protein at the cell surface compared with the cytosolic fraction. Experiments were performed using homogenates of whole cerebral and cremaster arteries. As shown in Figure 8A and B, in both vessel types more than 90% of total α-BK_Ca_ channels were located at the cell membrane. However, when cell surface expression of α-BK_Ca_ was normalized to equal amounts of α-smooth muscle actin in each preparation, cerebral arteries show approximately 20 times higher level of total amount of α-BK_Ca_ protein at the cell membrane compared with cremaster arteries (Figure 9).

## Discussion

In previous studies, we reported functional differences in BK_Ca_ channels in VSMCs between cerebral arteries and those from cremaster muscle [26], [32]. In those studies, we showed that BK_Ca_ from cremaster VSMCs exhibit a decreased Ca^2+^ sensitivity and suggested that this may, in part, be due to a decrease in the amount of the β1 regulatory subunit. Regulation of BK_Ca_ is, however, complex involving mechanisms at the levels of expression and post-translational modification, as well as its physical relationship to cellular organelles such as the sarcoplasmic reticulum [5], [6], [8]. On the basis of this, the aims of the present study were to examine whether differences exist in the expression of splice variants of the α-subunit of BK_Ca_ and if membrane location of the channel differed between the two vascular beds.

The major findings of the present studies were as follows: Firstly, qPCR studies demonstrate a significantly higher mRNA expression for the BK_Ca_ α-subunit STREX splice variant in rat cremaster arteries compared with that in cerebral arteries. Secondly, we detected the predominant cell surface expression of both the α-BK_Ca_ splice variants ZERO and STREX in cell expression systems, with no apparent impact of β1-subunit co-expression on the degree of cell surface localization. Thirdly, although the β1-subunit expressed alone is able to reach the cell membrane, a significant proportion remains cytosolic compared with its predominant cell surface localization in the presence of either the ZERO or STREX variant. Finally, cell surface labeling revealed that the vast majority of α-BK_Ca_ channel (>90%) in both cremaster and cerebral arteries is located in the cell membrane fraction under basal conditions. However, a major difference between the two vascular beds was that the total amount of plasma membrane α-BK_Ca_ is approximately 20 times lower in cremaster arterioles as compared with small cerebral arteries.

Although a single gene, KCNMA1, encodes the pore forming BK_Ca_ α-subunit in vertebrates, there is considerable phenotypic diversity of these channels in different tissues. Several factors are known to contribute to this diversity including, alternative splicing [36], the co-expression of regulatory β-subunits [37], [38], and post-translational modifications including protein phosphorylation [39]. Modulation of BK_Ca_ channels by a complex network of signal transduction pathways such as reversible protein phosphorylation has been studied extensively [39]–[41]. Importantly, alternate splicing of pre-mRNA leading to channel variants with differing degrees of modulation by reversible protein phosphorylation represents a potential mechanism to generate functional diversity of ion channels. In studies of cloned mouse BK_Ca_ variants, expressed in HEK293 cells, Tian et al. (2001) demonstrated that cAMP-dependent protein kinase (PKA)-mediated phosphorylation activates BK_Ca_ ZERO variant, but inhibits the STREX variant, which could thus impact channel function including Ca^2+^ sensitivity [17]. The level of STREX expression also has important modulatory consequences as it has been previously shown that only one subunit within the tetrameric holo-channel needs to be of the STREX type to alter channel function [42]. It has also been shown that protein palmitoylation (a post-translational modification affecting multiple ion channels) can regulate the activity and surface expression of BK_Ca_ channel α-subunits in native tissues and cultured cells [19]. Specifically, Tian and colleagues described palmitoylated BK_Ca_ channels that include plasma membrane associated STREX variants that are inhibited by PKA-dependent phosphorylation, whereas ZERO channels are activated by PKA [43]. Therefore, the finding of lower expression of the ZERO variant and higher expression of STREX in cremaster arteries compared with mid-cerebral arteries could conceivably relate to functional alterations in BK_Ca_ Ca^2+^ sensitivity, as we have previously observed for cremaster vascular smooth muscle cells [32].

However, in the present studies the cremaster vessels have been shown to express a higher level of STREX as compared to the cerebral arteries which may have been expected to convey a higher Ca^2+^ sensitivity [9] rather than the decreased Ca^2+^ sensitivity we previously reported [26]. This apparent discrepancy may relate to a number of factors including the dominant effects of differences in overall expression levels and also that the cremaster vessels were previously shown to have a lower ratio of β1:α subunit. Importantly, the β1 subunit has previously been shown to contribute to the Ca^2+^ sensitivity of the channel [44]. An additional consideration is that our measurements of STREX and ZERO expression were limited to the mRNA levels. In this previous study, we also reported a decrease in the ratio of BK_Ca_ α:β subunits in the crude membrane fraction of cremaster vessels compared with small cerebral arteries [26]. The data from the present study suggest it is unlikely that a lower level of β1-BK_Ca_ would negatively influence insertion of the α-subunit into the membrane, as our data in cell expression systems showed similar levels of surface location for α-BK_Ca_ in the presence and absence of the β1-subunit. Interestingly, and in contrast, the presence of the α-subunit in the expression system resulted in an increased proportion of β1-subunit being located at the cell surface.

In earlier studies, Jackson and Blair (1998) suggested that BK_Ca_ is ‘silent’ in cremaster muscle vasculature under basal conditions, but may be ‘recruited’ under stimulated conditions. Such stimulation was suggested to include vasoconstriction evoked by catecholamines and high tissue PO_2_ levels [27]. Whether such recruitment involves differences in splice variant expression, translocation of channel subunit proteins to the plasma membrane or post-translational modifications such as phosphorylation has not been fully elucidated. Given the high levels of α-subunit protein found at the membrane in both vessel types, it is unlikely that a simple difference in membrane vs. cytosolic pools explains the differences observed between cremaster and cerebral vessels. This does not, however, exclude the possibility that a dynamic alteration in channel protein trafficking occurs under other conditions.

In the present study, we also found a very low level of expression of the SS4 α-subunit variant relative to the ZERO variant in mid-cerebral, with no detectable expression in cremaster arteries. Although expression of the SS4 variant in vasculature (identified by RT-PCR) has been previously reported in cerebral and coronary arteries [11], its functional importance, particularly in native tissues such as small arteries, is unknown. Similarly, the functional significance of a lack of SS4 variant expression (as we report for cremaster muscle arterioles) is unclear. Using a *Xenopus* oocytes expression system, previous studies have suggested that ZERO and SS4 variants exhibit identical BK_Ca_ channel characteristics, including single-channel conductance and voltage dependent activation [18]. Those authors did, however, show that the activation rates of SS4 channels were more rapid at a similar voltage compared with the ZERO form when [Ca^2+^]_i_ was higher than 5 µM. In addition to a comparative lack of information as to any functional significance of very low expression levels of the SS4 variant (approximately 0.4% of total α-BK_Ca_ mRNA) in cerebral arteries, it should also be considered that non-smooth muscle cell contamination (for example from neurons or adventitial cells) in whole vessel preparations could contribute to this signal.

Apart from differences in splice variant expression, it would be expected that the marked difference in total BK_Ca_ channel protein expression would be of functional significance. This is despite the large conductance (approx 240 pS) in VSMCs from both vascular beds. Specifically, an ∼20-fold higher level of α-BK_Ca_ protein was detected in cerebral arteries compared with cremaster arteries. Importantly, this would be reflected at the plasma membrane because a similar proportion of total BK_Ca_ was surface located in both cerebral and cremaster arteries, as shown by our biotinylation assay. While measurements were performed on homogenates of whole vessels, the majority of signal would be expected to derive from the VSMC layers. Endothelial cells of healthy arteries are thought to be devoid of BK_Ca_ channels, although this point has been somewhat controversial [45]. Cellular capacitance measurements performed in our previous studies indicate that VSMC size in the two vessel types is similar, suggesting that functional effects of the expression difference would not be compensated by differences in size alone [26]. The earlier study also demonstrated, in crude membrane fractions, increased α-BK_Ca_ protein (approx. 3x) in cerebral vessels as compared to cremaster arterioles. Despite these measurements of marked differences in expression at the protein level actual K^+^ currents only differed by approximately 2x (at 5 mM Ca^2+^). Conceivably the seemingly disparate findings may relate to the functional status of the channels including as determined by post-translational modifications such as phosphorylation or possibly the influence of splice variants not directly examined in this study [46]. Another factor perhaps affecting the differences in absolute protein levels between cerebral vessels and cremaster arterioles relates to differences in adventitial structure that we have previously demonstrated [47]. It could be argued that these differences impact the access of the biotinylation reagents. We believe this to be unlikely, however, as the biotinylation reagents are small in regard to molecular weight and it was previously shown that the molecules easily penetrate the vascular wall [33]. Further, while this could theoretically effect the magnitude of the protein expression levels it would not impact the α to β subunit ratios nor the distribution between the plasma membrane and cytosol. As both the cell surface biotinylation approach and measurements performed in crude membrane fractions showed qualitatively similar same results, the conclusion that protein expression levels are greater in cerebral vessels appears robust.

In summary, significant differences exist with respect to the BK_Ca_ splice variants expressed in cremaster muscle arterioles compared with small arteries from the cerebral vasculature. Specifically, a higher expression level of the STREX variant of the α-subunit was observed in arterioles from cremaster muscle. While functional significance of this finding is yet to be fully demonstrated, it appears that it does not affect the plasma membrane location of the channels as >95% of α-subunit was found to be at the cell surface in both vessel preparations. In contrast, a marked difference in the detectable expression level was observed, with cerebral arteries expressing α-subunit protein at a level 20 times greater than that of cremaster arterioles.
